# Optimal Antithrombotic Treatment of Patients with Atrial Fibrillation Early after an Acute Coronary Syndrome—Triple Therapy, Dual Antithrombotic Therapy with an Anticoagulant… Or, Rather, Temporary Dual Antiplatelet Therapy?

**DOI:** 10.3390/jcm9082673

**Published:** 2020-08-18

**Authors:** Ugo Limbruno, Francesco De Sensi, Alberto Cresti, Andrea Picchi, Fabio Lena, Raffaele De Caterina

**Affiliations:** 1 Cardioneurovascular Department, Azienda USL Toscana Sudest, 58100 Grosseto, Italy; ugo.limbruno@uslsudest.toscana.it (U.L.); francesco.desensi@uslsudest.toscana.it (F.D.S.); alberto.cresti@uslsudest.toscana.it (A.C.); andrea.picchi@uslsudest.toscana.it (A.P.); 2 Pharmacy Department, Azienda USL Toscana Sudest, 58100 Grosseto, Italy; fabio.lena@uslsudest.toscana.it; 3 Cardio-Thoracic and Vascular Department, Pisa University Hospital and University of Pisa, 56124 Pisa, Italy; 4 Fondazione VillaSerena per la Ricerca, Città Sant’Angelo, 65013 Pescara, Italy

**Keywords:** acute coronary syndrome, atrial fibrillation, oral anticoagulant therapy, antithrombotic therapy, P2Y_12_ inhibition, tailored therapy

## Abstract

The combination of atrial fibrillation (AF) and acute coronary syndrome (ACS) is a complex situation in which a three-dimensional risk—cardioembolic, coronary, and hemorrhagic—has to be carefully managed. Triple antithrombotic therapy (TAT) is burdened with a high risk of serious bleeding, while dual antithrombotic therapy with an anticoagulant (DAT) likely provides only suboptimal coronary protection early after stent implantation. Moreover, TAT precludes the advantages provided by the use of the latest and more potent P2Y_12_ inhibitors in ACS patients. Here, we aimed to simulate and compare the expected coronary, cardioembolic, and hemorrhagic outcomes offered by DAT, TAT, or modern dual antiplatelet therapy (DAPT) with aspirin plus one of the latest P2Y_12_ inhibitors in AF patients early after an ACS. The comparison of numbers needed to treat to prevent major adverse events with the various antithrombotic regimens suggests that AF–ACS patients at high ischemic and hemorrhagic risk and at moderately low embolic risk (CHA_2_DS_2_VASc score 2–4) might safely withhold anticoagulation after revascularization for one month taking advantage of a modern DAPT, with a favorable risk-to-benefit ratio. In conclusion, this strategy, not sufficiently addressed in recent European and North American guidelines or consensus documents, adds to the spectrum of treatment options in these difficult patients; it might be the best choice in a substantial number of patients; and should be prospectively tested in a randomized controlled trial.

## 1. Introduction

The combination of atrial fibrillation (AF) and acute coronary syndrome (ACS) poses complex therapeutic dilemmas. Here, cardiologists face a three-dimensional risk—cardioembolic, coronary, and hemorrhagic—which must be carefully managed and with important threats. Triple antithrombotic combination therapy (TAT), i.e., oral anticoagulation (OAC) on top of dual antiplatelet therapy (DAPT), has been for two decades the milestone for treatment of these patients. Due to the high and sometimes prohibitive hemorrhagic burden of TAT, several dual antithrombotic therapy (DAT) regimens, i.e., OAC plus a single antiplatelet agent, have been proposed in randomized trials [1,2,3,4,5] with the aim of improving safety without hampering efficacy. Current guidelines [1,2] endorse this approach in patients at high bleeding risk. In such a condition, OAC has, however, always remained an undisputed cornerstone.

We here describe the rather frequent scenario of a patient admitted to our hospital, in order to argue against such a mantra, advocating the possibility of temporarily omitting anticoagulation in some circumstances.

## 2. Case Vignette

**Figure 1:**
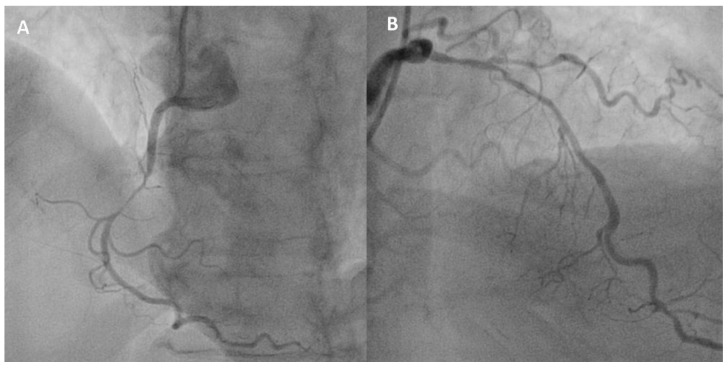
Coronary angiography in the patient case described in the case vignette. (**A**) The right coronary artery shows a tight stenosis in the mid segment (culprit lesion). (**B**) The left anterior descending coronary artery shows a long 75% maximum diameter stenosis in the proximal and mid segments.

A 73 year-old male with a history of hypertension and AF in treatment with rivaroxaban 20 mg/day was admitted to our hospital for a non-ST segment elevation ACS. The electrocardiogram (ECG) on admission showed AF and ST-segment depression in the inferior leads. Coronary angiography revealed a tight stenosis in the right coronary artery and a long 75% maximum diameter stenosis in the proximal and mid-left anterior descending coronary artery (Figure 1).

**Figure 2:**
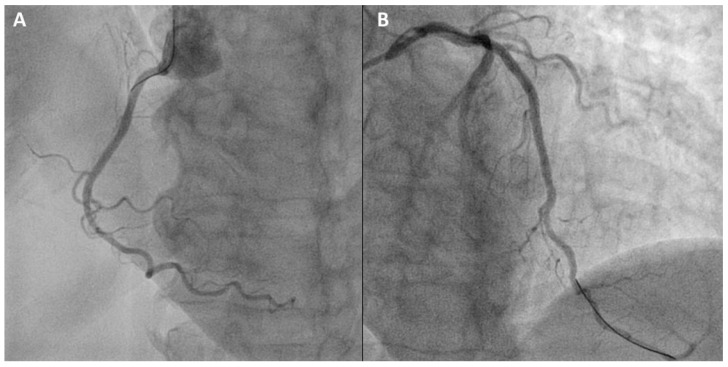
Percutaneous coronary intervention in the patient case described in the case vignette. (**A**) The right coronary artery lesion was treated with the implant of a Xience Prime (2.75 × 28 mm) stent. (**B**) The proximal and mid left anterior descending coronary artery lesions were treated with the implantation of three Xience Prime (3.0 × 38 mm, 3.0 × 15 mm, 2.75 × 15 mm) stents.

His CHA_2_DS_2_VASc score was 3 (age 65–74, hypertension, vascular disease). Because of this, OAC was considered necessary. The patient received percutaneous coronary intervention (PCI) of the right coronary artery, considered the culprit vessel, with a Xience Prime (2.75 × 28 mm) stent (Figure 2A). Three days later, the proximal and mid left anterior descending coronary artery lesions were treated with the implantation of three Xience Prime (3.0 × 38 mm, 3.0 × 15 mm, 2.75 × 15 mm) stents (Figure 2B).

After the procedure, TAT with aspirin, clopidogrel, and rivaroxaban 15 mg/day was prescribed. However, because of the patient’s high bleeding risk (HAS-BLED) score (hypertension, age, abnormal renal function, need for antiplatelet therapy = 4), and because blood tests on day four showed a drop of hemoglobin from an initial value of 12.0 to 8.5 g/dL, aspirin was discontinued on day six, and the patient was discharged on a combination of rivaroxaban 15 mg/day and clopidogrel 75 mg/day.

**Figure 3:**
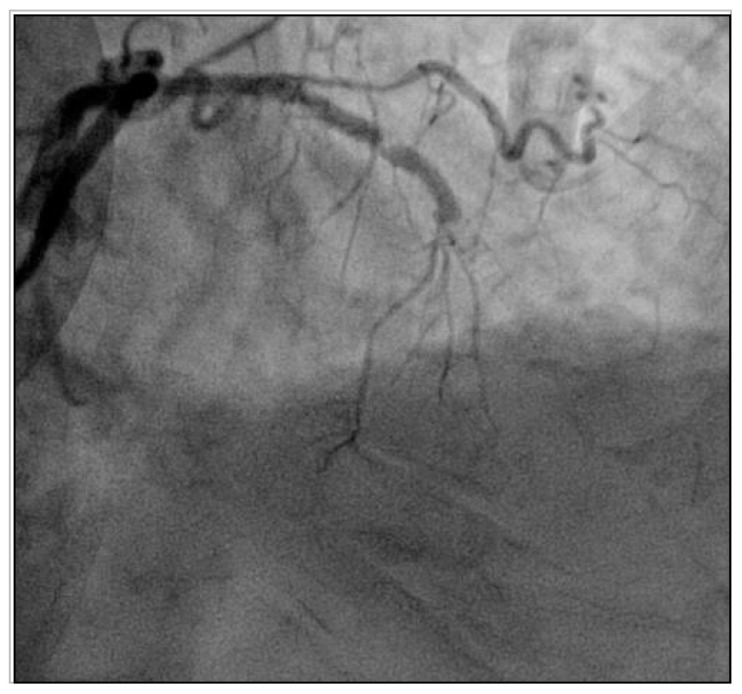
Coronary angiography at readmission in the patient case described in the case vignette. Subacute stent thrombosis, with a total vessel occlusion in the left anterior descending coronary artery three days after the percutaneous coronary intervention (PCI) illustrated in Figure 2.

Two weeks after the hospital discharge, the patient was readmitted to the emergency department, this time with an anterior ST-segment elevation myocardial infarction. Coronary angiography showed a subacute stent thrombosis of the left anterior descending coronary artery (Figure 3), which was effectively treated with mechanical thrombus aspiration and balloon dilatation.

On the assumption that the combination of rivaroxaban 15 mg/day and clopidogrel had proved inadequate to prevent ischemic events and stent thrombosis in the first weeks after stent implantation, DAPT consisting of aspirin 100 mg/day and ticagrelor (loading dose 180 mg followed by 90 mg twice daily (bid)) was enacted, and maintained up to one month, dropping OAC. Subsequently, the previous combination (rivaroxaban 15 mg and clopidogrel instead of ticagrelor) was re-installed. There were no further sequelae, and the patient recovered uneventfully.

## 3. Case Discussion

The 2018 Joint European Society of Cardiology (ESC) consensus document on the management of antithrombotic therapy in AF patients presenting with ACS [6] recommends, as do previous ESC guidelines and a recent scientific statement from the American College of Cardiology (ACC)/American Heart Association (AHA) [7], decisional algorithms by which the intensity and duration of OAC plus antiplatelet therapy are exclusively based on the assessment of cardiovascular (ischemic) and bleeding risks. Here, cardioembolic risk, estimated with the CHA_2_DS_2_VASc score, is considered in practice as an all-or-none categorical variable: namely, AF patients with a CHA_2_DS_2_VASc score ≥2 who develop an ACS or, conversely, ACS patients who develop AF, have a strict indication to OAC. However, this CHA_2_DS_2_VASc score threshold is borrowed from studies evaluating large AF populations mostly without an ACS. These latter patients are, in principle, also in need for concomitant antiplatelet therapy, especially after receiving coronary stenting, now a routine option in such patients [6]. The addition of antiplatelet therapy to OAC significantly increases the hemorrhagic risk, with a debatable addition to cardioembolic protection. This inevitably changes the bleeding profile of the patient and should entail a shift in the CHA_2_DS_2_VASc score level at which OAC addition becomes favorable compared to its risk. Moreover, current guidelines weigh the ischemic risk only in relation to the duration of TAT or DAT including an anticoagulant, and do not sufficiently consider the lower potency, and therefore the changed tradeoff in terms of cardiovascular risk, deriving from the use of clopidogrel instead of the latest P2Y_12_ inhibitors, as well as from aspirin withdrawal.

We here challenge the currently claimed lack of alternatives to the “anticoagulant always” approach, with the following questions:

- Do we really know the tradeoff (benefit vs. risk) in such patients for a strategy based on early OAC initiation or continuation soon after revascularization in AF–ACS patients, especially with a low CHA_2_DS_2_VASc score and a high coronary risk, compared with a DAPT-only strategy?
- Should we then apply the same “in-or-out” thresholds for an obligatory anticoagulant prescription derived from non-ACS populations to such a specific clinical setting, even in the early phases after a coronary stenting?
- Should it not be reasonable to withhold OAC for some time, e.g., for the first month after revascularization in such AF–ACS patients, especially now that prasugrel and ticagrelor are widely available and known to be superior to clopidogrel in preventing atherothrombotic events and stent thrombosis [8,9]?

## 4. Prevention of Cardioembolic Stroke and Systemic Embolic Events

**Figure 4:**
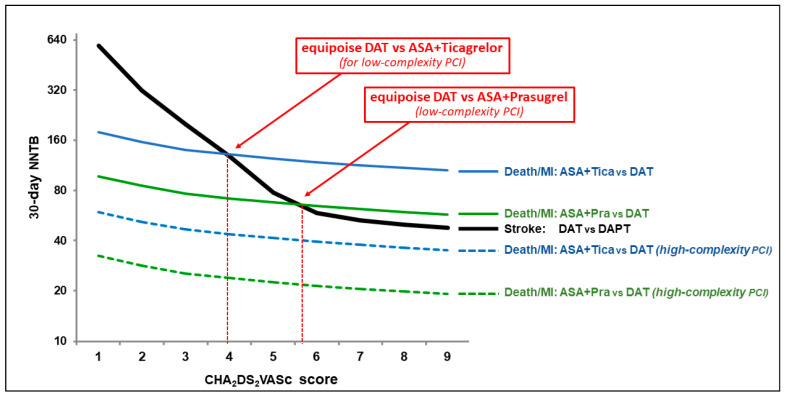
Calculated comparative numbers needed to treat and to prevent stroke or coronary events at 30 days for various antithrombotic drug combinations in patients with atrial fibrillation and a recent coronary stenting for an acute coronary syndrome. NNTBs for coronary events deriving from the use of DAPT with ticagrelor instead of DAT with clopidogrel appear to be lower than the NNTBs for stroke deriving from the use of DAT instead of DAPT for CHA_2_DS_2_VASc scores < 4. NNTBs for coronary events deriving from the use of DAPT with prasugrel instead of DAT with clopidogrel appear to be lower than the NNTBs for stroke deriving from the use of DAT instead of DAPT for CHA_2_DS_2_VASc scores <5. In case of high-complexity PCI, the event ratio favors DAPT with both ticagrelor and prasugrel at any CHA_2_DS_2_VASc score. See the Online Appendix of [15] for calculations of different 30-day NNTBs comparing different antithrombotic strategies. Complex PCI is defined as at least three of the following features: long, bifurcated, eccentric, thrombotic, tortuous, angulated or calcified lesions, left main PCI, and multiple vessel PCI [16]. Abbreviations: ACS: acute coronary syndrome; DAPT: dual antiplatelet therapy (aspirin + clopidogrel); DAT: dual antithrombotic therapy (an anticoagulant plus a P2Y_12_ inhibitor antiplatelet therapy); NNTB: number needed to treat for benefit; OAC: oral anticoagulation; PCI: percutaneous coronary intervention.

Oral anticoagulation is the well-established gold standard for prevention of cardioembolic events in AF patients [10,11]. The yearly risk of cardioembolism in non-anticoagulated AF patients with CHA_2_DS_2_VASc scores of 2 or 3 has been estimated to be in the order of 3.7 and 5.9/100 patients, respectively [12,13]. The monthly-based risk of stroke or systemic embolic events in these patients can be estimated—although with some uncertainty due to the lack of specific data—dividing the yearly risk by 12, therefore at levels of 0.3, 0.5 and 0.8/100 patients, respectively. During the early days after an ACS, these rates are likely to be increased, and it has been reported that this occurs by a factor of 3.9 [14]. Taking into account this ACS-related increase in the risk of stroke and assuming a 64% lower risk with full anticoagulation [10] and even a 72% higher risk with dual antiplatelet therapy (DAPT) with aspirin and clopidogrel in comparison to full anticoagulation [11], the number of patients needed to treat for benefit (NNTB) with OAC in addition to DAPT with aspirin and clopidogrel to prevent an ischemic stroke or a systemic embolic event during the first month after an ACS was calculated as previously described [15]: in the case of CHA_2_DS_2_VASc = 2, the NNTB would be a non-negligible number of 320 patients (201 patients in case of CHA_2_DS_2_VASc = 3) (see Figure 4 for NNTBs in patients with higher CHA_2_DS_2_VASc score).

Several recent observational data question the effectiveness of OAC early after an ACS in properly treated patients with a relatively low CHA_2_DS_2_VASc score in whom OAC is currently recommended. Indeed, in two consecutive series of AF patients receiving drug-eluting stent implantation, DAPT alone was not associated with a significant increase in the risk for ischemic stroke compared to TAT [17,18]. Moreover, DAPT yielded a highly significant reduction in the risk of intracranial hemorrhage, although this finding might have been amplified by the use of warfarin instead of NOAC in the TAT group [17,18]. In a recent claim-based large observational study, the benefit of OAC in AF patients with a CHA_2_DS_2_VASc score 2–4 and a CHADS_2_ score <2 before the use of OAC did not result in a lower composite risk of stroke, systemic embolism, and death (hazard ratio, HR: 1.00), yet it was associated with a higher risk of bleeding (HR: 1.70, 95% confidence interval (CI), 1.46–1.97) [19]. Finally, a temporary suspension of oral anticoagulation has been proved reasonably safe in some clinical settings: in the BRIDGE trial, patients undergoing invasive procedures who discontinued warfarin with no bridging with dalteparin did not show any increase of thromboembolic events despite almost 10 days of likely sub-therapeutic International Normalized Ratio (INR) [20].

Thus, a temporary withholding of OAC in AF–ACS patients with a low CHA_2_DS_2_VASc score for up to four weeks after an ACS appears to be reasonable as to the occurrence of cardioembolic events, considering the concomitant use of DAPT, which has been demonstrated to be somewhat more effective than aspirin alone, although not as OAC, in reducing stroke risk [8]. As an additional consideration, recent findings suggest that the use of new P2Y_12_ inhibitors instead of clopidogrel might further increase the effectiveness of DAPT in preventing cardioembolic stroke [21,22], thus further reducing the efficacy gap on this specific endpoint between OAC in the setting of TAT vs. DAPT when one of the new P2Y_12_ inhibitors is used.

## 5. Prevention of Coronary Ischemic Events

If a strategy of triple therapy is chosen in patients with concomitant AF and ACS, current guidelines strongly recommend against the use of the new P2Y_12_ inhibitors, the current standard of care after an ACS without AF. Ticagrelor and prasugrel have been clearly demonstrated to reduce the rate of cardiovascular events (cardiovascular death, reinfarction, and stroke) when compared to clopidogrel in the PLATO and TRITON-TIMI 38 trials, with a risk reduction within the first month of treatment ranging from 12% (ticagrelor) [8] to 22% (prasugrel) [9]. Since the incidence of cardiovascular events after ACS is also related, as is the embolic risk, to the CHA_2_DS_2_VASc score [23,24], we can expect a 30-day NNTB for cardiovascular events ranging from 179 (CHA_2_DS_2_VASc = 1) to 105 (CHA_2_DS_2_VASc = 9) with the use of ticagrelor instead of clopidogrel and from 97 (CHA_2_DS_2_VASc = 1) to 57 (CHA_2_DS_2_VASc = 9) with the use of prasugrel instead of clopidogrel (Figure 4; see Online Appendix of [15] for calculations). The calculated equipoise between NNTBs for stroke versus coronary events is at CHA_2_DS_2_VASc = 4 for DAPT with ticagrelor instead of DAT and at CHA_2_DS_2_VASc = 5 for DAPT with prasugrel instead of DAT (Figure 4). In patients with high-risk target lesions [16,25,26], the rate of early cardiovascular events, including stent thrombosis, is further increased and, accordingly, the above mentioned NNTBs deriving from the use of the new P2Y_12_ inhibitors instead of clopidogrel (the recommended P2Y_12_ inhibitor in case of TAT) are expected to be even lower. A 3.4 HR for early stent thrombosis is expected to occur in clopidogrel-treated ACS patients with ≥ 3 compared to one high-risk procedural feature among the following: long, bifurcated, eccentric, thrombotic, tortuous, angulated or calcified lesions, left main and multiple vessel PCI [16]. As a consequence, a 70% further reduction in the above-mentioned NNTBs has to be expected in case of high-complexity PCIs when modern DAPT with ticagrelor or prasugrel is used instead of DAT (Figure 4).

Based on the results of RE-DUAL PCI [2], PIONEER-AF [3], AUGUSTUS [4], and ENTRUST-AF PCI [5] studies, and on two recent meta-analyses [27,28], a dual-pathway antithrombotic therapy (DAT) using a single antiplatelet agent (a P2Y_12_ inhibitor) is now recommended in most AF patients early after PCI. Although such studies have shown a low absolute incidence of stent thrombosis, they included only a fraction of patients with ACS (ranging from 31 to 62% in different trials), and were largely underpowered to truly assess efficacy in terms of cardioembolic or coronary events, making the confidence about the beneficial net clinical benefit of this strategy in terms of protection from coronary events and stent thrombosis in ACS patients questionable, especially in those submitted to high-complexity PCI. Indeed, the RE-DUAL PCI study actually showed a trend towards a higher rate of myocardial infarction and stent thrombosis in the subgroup of patients treated with dabigatran 110 mg twice daily + a single antiplatelet drug with a P2Y_12_ inhibitor, as compared to a “classical” TAT strategy including warfarin, aspirin, and a P2Y_12_ inhibitor [2] (this was not apparent, however, in the dabigatran 150 mg twice daily + single antiplatelet drug) [2]. In PIONEER-AF, the dual therapy arm (rivaroxaban 15 mg once daily + a P2Y_12_ inhibitor) showed a numerically higher rate of cardiovascular events in the subgroup of patients undergoing PCI on bifurcation lesions, as compared to the classical TAT arm including warfarin [3]. In AUGUSTUS, the risk of coronary events—including myocardial infarction, urgent revascularization, and stent thrombosis—was numerically higher (although not significantly) in the two study arms in which aspirin was omitted as compared to the TAT arms, and the incidence of stent thrombosis was nearly twice as high [4] and, interestingly, almost exclusively confined to the first month after PCI [29]. A similar trend was finally observed in the recent ENTRUST-AF PCI trial where a 37% increase in definite stent thrombosis and a 24% increase in mortality occurred in the dual combination therapy arm [5]. In a recent meta-analysis [27] including the RE-DUAL PCI [2], PIONEER-AF [3], AUGUSTUS [4], and ENTRUST-AF PCI [5] trials, a trend towards a 55% higher stent thrombosis was calculated with the use of DAT compared to warfarin-based TAT. This increase in stent thrombosis did not attain conventional statistical significance (*p* = 0.06), but the meta-analysis population size, about 4000 patients per arm, was admittedly largely underpowered to clarify this point. Indeed, the sample size required to detect a 55% increase with an event rate of 0.7% per year at a two-sided significance level of 0.05 with 90% power would have to include at least 10.500 patients per arm. Moreover, the TAT used as a reference in these meta-analyses [27,28] cannot be considered as the state-of-the-art for coronary protection, since it precludes the use of the new P2Y_12_ inhibitors, and is inevitably burdened by a high incidence of hemorrhages. What if DAT were to be compared to the current state-of-the-art DAPT (aspirin + one of the new P2Y_12_ inhibitor) instead of TAT, especially in the case of high-risk PCI?

## 6. Bleeding Risk with Differing Antithrombotic Strategies

The risk of bleeding events is highest during the first weeks after initiation of any antithrombotic treatment [30]. At the same time, the highest risk for coronary ischemic events in case of bleeding-induced discontinuation of antiplatelet agents is in the days and weeks after the index event [31]. Therefore, the choice of a balanced antithrombotic strategy in the first weeks after an ACS is of utmost importance to avoid major bleeding events.

**Figure 5:**
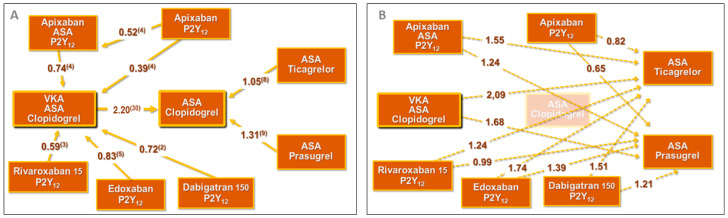
Bleeding hazard ratios for different antithrombotic treatments. (**A**) Direct comparisons from randomized trials or observational studies. References are reported for each hazard ratio. (**B**) Indirect network comparisons of different antithrombotic treatments.

Since any direct comparison between combination therapies (various non-vitamin K antagonist oral anticoagulants (NOACs) against warfarin on top of single or dual antiplatelet regimen) and current state-of-the-art DAPT (aspirin plus ticagrelor/prasugrel) is not available, we cannot currently decide which of these antithrombotic strategies is safer with regard to bleeding. However, we may attempt an exploratory indirect comparison through an indirect network analysis of observational and randomized studies comparing different antithrombotic strategies. From the large National Patient Registry of Denmark [30], there is an estimate that warfarin-based TAT carries a 2.2 adjusted HR for early bleeding when compared to DAPT with clopidogrel in AF patients undergoing revascularization. At the same time, several randomized trials have provided HRs for bleeding between different dual-pathway combination therapies as compared to warfarin-based TAT [1,2,3,4,5] and, on the other hand, for “newer” DAPT as compared to “older” DAPT [8,9] (Figure 5A). The indirect network comparisons, derived from these HRs, are reported in Figure 5B.

This network analysis suggests that (a) any TAT exposes patients to a higher risk of bleeding with respect to any “newer” or “older” DAPT; (b) DAT with dabigatran 150 mg BID entails a higher bleeding risk compared to both ticagrelor- and prasugrel-based DAPT regimens (HR 1.51 and 1.21, respectively); and (c) a DAT with rivaroxaban 15 mg would feature a higher bleeding risk with respect to ticagrelor-based DAPT (HR 1.24) and a similar bleeding risk with respect to prasugrel on top of aspirin. On the contrary, the network analysis suggests a slightly safer profile for DAT with apixaban 5 mg BID compared to any DAPT (HRs 0.86, 0.82, and 0.65 when compared to DAPT with clopidogrel, ticagrelor, or prasugrel, respectively). The higher risk of bleeding with edoxaban 60 mg/day + clopidogrel compared with the classical TAT with warfarin, aspirin, and clopidogrel in ENTRUST-AF PCI in the early time period after stenting, when warfarin anticoagulation in the TAT arm was documented to be suboptimal, would also argue for a higher HR for bleeding for an edoxaban-based DAT compared with a DAPT (which was the therapy practically in use in such early phases of TAT) [5].

## 7. Other Supporting Clues for an Early DAPT Strategy from ENTRUST-AF PCI

An analysis of the bleeding and ischemic outcomes of the recently reported ENTRUST-AF PCI [5] appears to further support our contention. Early after stenting, patients in the edoxaban–clopidogrel arm of the study experienced not only a numerical increase in bleeding—explained, as detailed above, by the suboptimal anticoagulation in the comparator TAT arm, but also a higher rate of ischemic events (the composite of cardiovascular death, stroke, systemic embolic events, myocardial infarction, and definite stent thrombosis). Due to ineffective warfarin anticoagulation early on in the study, this early time period can reasonably be assimilated to a comparison between edoxaban + clopidogrel DAT vs. aspirin + clopidogrel DAPT. It would thus appear that—in the overall AF patients included in the study—an early course of DAPT would be clearly of net clinical benefit compared with the tested DAT, being associated with less bleeding and also—quite interestingly—less ischemic events. In summary, a joint evaluation of the risk for cardioembolic stroke, coronary events, and bleeding in at least some patients with AF and recent PCI for ACS would appear to favor a modern DAPT instead of either TAT or DAT, with modern DAPT probably slightly inferior in protection from stroke, but superior in protecting from coronary events (most likely stent thrombosis).

## 8. What to Do Then When a High Coronary Risk Occurs with a Low Cardioembolic Risk? A Role for Modern DAPT First, and DAT Later?

Pre-existing or new-onset AF occurs in about 6% of patients undergoing PCI [32]. Current guidelines recommend the early start of TAT, preferably with a NOAC, in all AF–ACS patients at high ischemic risk for all patients with CHA_2_DS_2_VASc score >1 [6,7]. An early switch from TAT to DAT is then recommended in case of high bleeding risk. Unfortunately, as described in the case vignette illustrated above, a high ischemic risk often associates with a high bleeding risk, making both TAT and DAT inadequate to cover the entire spectrum of patients’ risk. As recommended by guidelines, we treated our patient with rivaroxaban 15 mg early after PCI and switched him from TAT to DAT at discharge. Our DAT strategy, according to the above mentioned calculations, theoretically allows to avert one stroke every 201 patients per month of treatment with an OAC instead of DAPT, but—at the same time—entails a risk of one additional coronary event every 140 or 77 patients per month of treatment with clopidogrel instead of ticagrelor or prasugrel, respectively, possibly plus a supplementary risk induced by aspirin withdrawal (Figure 4), and to a 24% higher rate of bleeding events as compared to DAPT with ticagrelor (Figure 5B). Our patient would have likely escaped the here-reported serious coronary event if he would have been treated by state-of-the-art DAPT (i.e., aspirin plus ticagrelor or prasugrel) in the first month after discharge, while the deferral of OAC initiation to after this critical time would have exposed him to just a trivial increase in the stroke risk.

## 9. Limitations

The main limitation of the present study is the total absence of randomized data comparing modern DAPT versus any dual pathway antithrombotic regimen in AF–ACS patients. Secondly, the relationship between the CHA_2_DS_2_VASc score and stroke risk in AF–ACS patients during the first 30 days after coronary event is likely different from patients with AF alone. According to [14], we conservatively calculated an HR of 3.9 for early stroke risk in AF–ACS patients with respect to AF-alone patients with the same CHA_2_DS_2_VASc score, although a large fraction of these early strokes in AF–ACS patients might be procedure-related and/or atheroembolic in origin. Finally, the best time window to withdraw OAC in favor of modern DAPT in these patients is purely speculative. In the ENTRUST trial, the analysis of the events early after randomization suggests that the introduction of OAC in the first 15 days after PCI might be counterproductive [33]. Meanwhile, the analysis of coronary events early after AUGUSTUS trial randomization to aspirin vs. placebo [29] suggests that full platelet inhibition is advisable for at least 30 days, the timepoint we suggest in the present viewpoint.

## 10. Conclusions

TAT is burdened with a high risk of serious bleeding early after its initiation and precludes the advantages provided by the use of the latest P2Y_12_ inhibitors. Current DAT likely provides only suboptimal coronary protection early after stent implantation, especially in cases of high-complexity PCI. Therefore, both treatments expose patients to the risk of major adverse events in the most critical time window, i.e., immediately after ACS. The critical AF–ACS patients, most likely those at high ischemic risk and at moderately low embolic risk (CHA_2_DS_2_VASc score 2–4), might safely withhold anticoagulation after revascularization for one month taking advantage of a modern DAPT with one the latest P2Y_12_ receptor inhibitors, with a favorable risk-to-benefit ratio. This strategy, not yet sufficiently addressed in recent European and North American guidelines and Consensus documents, adds to the spectrum of treatment options, and might be the best choice in a substantial number of these difficult patients.
